# Filler Models Revisited: Extension of the Nielson Model with Respect to the Geometric Arrangements of Fillers

**DOI:** 10.3390/polym14163327

**Published:** 2022-08-16

**Authors:** Johannes Macher, Pouyan Golestaneh, Astrid E. Macher, Matthias Morak, Andreas Hausberger

**Affiliations:** Polymer Competence Center Leoben GmbH, Roseggerstraße 12, A-8700 Leoben, Austria

**Keywords:** permeation, polymer, filler models, Nielsen, simulation

## Abstract

Models describing how fillers affect the barrier properties of polymers remain an important research topic to improve applications such as hydrogen storage or food preservation. The Nielsen model, one of the earliest models for such predictions, is still one of the most widely used in the literature. However, it does not provide quantitative information on arrangements of fillers inside a polymer matrix, which is crucial for the definition of suitable filler distributions in barrier materials. Therefore, the channel model was developed in this work, which extends the Nielsen model by determining the relative distances between the fillers in regular filler arrangements in polymer matrices. This allows us to relate the permeation properties of filled polymer membranes to the geometric properties of the filler arrangement in simulations and experimental measurements. Simulations with geometries defined according to the channel model showed good agreement with the predictions of the Nielsen model. This demonstrated that the channel model can be a valuable tool for predicting at least mean geometric distances in studied polymer membranes. The validity range of the channel model was limited to a value range of the filler volume fraction $0.01\leq \phi_{f}\leq 0.5$ based on theoretical considerations.

## 1. Introduction

Recently, the importance of polymers as barrier materials has steadily increased, e.g., hydrogen storage solutions [1,2,3], in food packaging [4,5], or solar cells [6,7]. For hydrogen applications, suitable materials are needed to increase the energy density of the hydrogen storage solution, to enhance hydrogen technology as an attractive alternative to battery or combustion systems. Among the many known storage concepts for hydrogen, high-pressure gas storage remains one of the storage solutions with a good weight-to-energy ratio. Cost-effective high-pressure hydrogen vessels with low weight and sufficient barrier properties are only possible by replacing the commonly used metallic liners with polymeric materials, since the use of polymers leads to simplified manufacturing [8].

Fillers are commonly used in polymeric materials to improve several material characteristics, e.g., mechanical, thermal, conductive, or barrier properties [3]. To investigate the barrier properties of filled polymer systems, several analytical models have been developed in recent decades to evaluate the influence of fillers on permeation properties based on only a few filler parameters, such as shape, volume fraction, or arrangement [9,10,11,12]. These models are only valid with certain constraints and assumptions about the fillers in the polymer matrix. In Table 1, the main properties and disadvantages of selected analytical models are listed. In this context, many models describe permeation through filled polymer matrices in 2D filler arrangements either by assuming that one dimension of the fillers is infinitely long (e.g., [13,14,15,16]) or by restricting the filler arrangements to two dimensions (e.g., [17,18]), while only a few analytical models describe permeation in 3D filler arrangements (e.g., [19,20]). Wolf et al. [10] even showed that analytical models agree with experimental data only moderately because of such limitations. Nevertheless, it is interesting that even simple analytical filler models, such as the Nielsen model with only two model parameters [13] or the Nielsen model modified by Bharadwaj et al. [15] with only three model parameters, showed good agreement with permeation experiments compared to other models (e.g., [21,22,23,24]). The reason for the good correspondence of such models without obvious complexity is not clear. Further research in analytical models is necessary to improve the understanding of permeation behavior in filled polymers and moreover to optimize the models.

Numerical simulation is an excellent tool for the prediction and computer-assisted modeling of physical processes and thus can be helpful in the development of improved permeation models. Zid et al. [11] gave a review of numerical models for permeation in filled polymers in 2D and 3D. Few publications discuss direct comparisons between simulation and analytical models, although some publications showed comparisons between numerical simulations and the Cussler and Aris models (e.g., [11,28,29]). Comparisons between numerical simulations and the Nielsen model or Nielsen-based models could not be found in the literature at all. We reasoned that too few model parameters are used in the Nielsen model, so that the geometry of filler arrangements in simulations cannot be constructed unambiguously. Nevertheless, such simulations could be a good basis for studies and improvements of analytical permeation models for filled polymers. It seemed necessary to develop an auxiliary model for the Nielsen model to provide the necessary geometrical constraints for suitable simulation setups.

Therefore, the main contributions of this work are as follows.

1. The derivation of a model as a supplement to the Nielsen model, which is called the “channel model”. The name was chosen because it is based on the assumption that channels with no inhibition of permeation due to fillers will form in a regular filler arrangement under certain conditions. This auxiliary model provides additional constraints for 2D FEM simulations with regular filler arrangements. No reference to a similar concept could be found in the literature.
2. The comparison of exemplary numerical simulations over wide ranges of filler volume fractions and aspect ratios with the predictions shown by the Nielsen model.
3. The discussion of additional constraints on the geometrical parameters of the setup to ensure the validity of the channel model.

## 2. Theoretical Background

### 2.1. Permeation Theory for Dense Polymer Membranes

**Figure 1:**
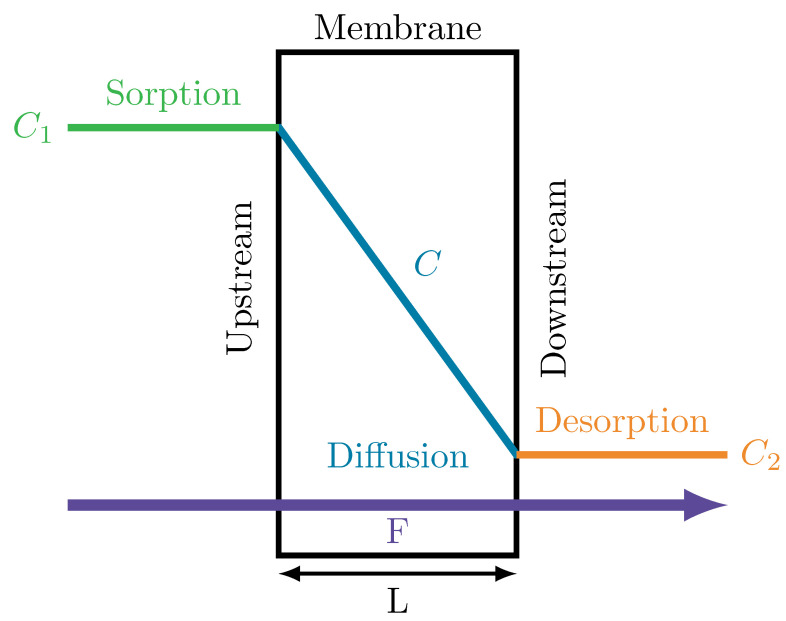
Sketch of the three-step process of mass permeation flow *F* through a dense polymer membrane with thickness *L*: sorption at the upstream side of the membrane with $C_{1}$ as boundary condition for Equation (4), diffusion through the membrane with the concentration distribution *C* inside the membrane, and desorption at the downstream side of the membrane with $C_{2}$ as the boundary condition.

Mass permeation of gas molecules through dense polymer membranes is generally described as a three-step process (see Figure 1) [30,31]:

1. Sorption on the upstream surface (high partial gas pressure of permeate);
2. Mass diffusion through the polymer membrane;
3. Desorption from the downstream surface (low partial gas pressure of permeate).

Thereby, it is assumed that the sorption and desorption processes are significantly faster than the mass diffusion, leading to fast thermodynamic equilibria on the surfaces, which is described with the solubility coefficient *S* to calculate the local permeate concentration *C* with

(1) C=S(p,T)·p

where *p* is the local partial gas pressure of the permeate and *T* is the ambient temperature. When the gas molecules are small and the application temperature is above the glass transition temperature of the polymer matrix, which is often the case for elastomers and semi-crystalline thermoplastics commonly used for liners and sealings in hydrogen storage solutions [8], the Henry model, where the solubility coefficient *S* is constant, can be applied [31].

Mass diffusion is a kinetic process where small molecules are transferred due to their random movements, resulting in a net flow from locations with high molecular concentrations to those with low concentrations [31,32]. This process is described with the comparatively simple Fick’s First Law:

(2) F=−D∇C

where the flow density *F* is related to the diffusion coefficient *D* and the spatial gradient of the concentration ∇C. In this work, only the Henry model is considered for Equation (1), which allows the modification of Equation (2) to

(3) $$F=-\underset{Pe}{\underbrace{D\;S}}\;\nabla p.$$

Equation (3) is one way to introduce the permeation coefficient Pe as the product of the solubility coefficient *S* and the diffusion coefficient *D*.

Fick’s Second Law,

(4) $$\frac{\partial C}{\partial t}=D\;\Delta C$$

which describes the transient development of the concentration within the membrane during the permeation process, is derived from Equation (2). In this case, the diffusion coefficient *D* is considered constant, so only the partial derivatives of concentration *C* appear in the equation. Detailed discussions of analytical solutions of Equation (4) in 1D can be found in [32,33]. When steady state is reached (∂C/∂t=0), Equation (4) reduces to the well-known Laplace equation

(5) ΔC=0.

Its solution, applied in Equation (3), allows the calculation of the permeation flow *F* in steady state.

### 2.2. Derivation of the Nielsen Model for Filled Polymers

**Figure 2:**
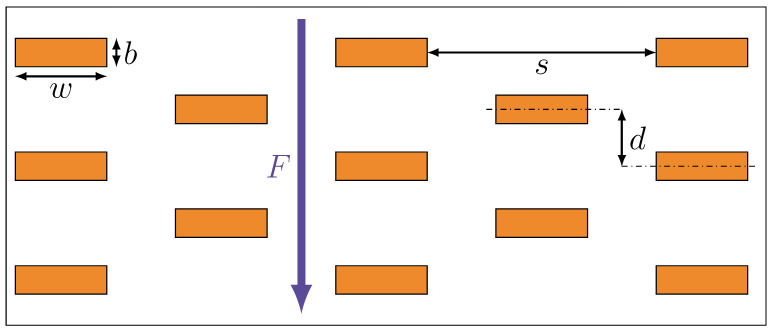
Schematic diagram for fillers in a regular arrangement and important filler parameters (filler width *w*, filler thickness (breadth) *b*, filler row distance *d*, slit distance *s*, and permeation flow *F* (main direction of diffusion)).

In the Nielsen model, the fillers are regularly and periodically arranged in a 2D polymer matrix. The fillers are defined as ribbons with infinite length and are represented in the 2D model as rectangular, with the long edge (filler width *w*) perpendicular to the main diffusion direction (see Figure 2). Due to the 2D representation of the polymer matrix and the small thickness of membranes compared to their lateral dimensions, diffusion is considered as a 1D problem. This approximation is, for example, sufficiently accurate for disk-shaped membranes with radii at least five times larger than the thickness [32]. For 1D problems and assuming steady state, Equation (3) changes to

(6) $$F=-\underset{Pe}{\underbrace{D\;S}}\;\frac{p_{1}-p_{2}}{L}$$

with the partial pressures $p_{1}$ and $p_{2}$ of the permeating gas at the upstream and downstream side, respectively, and the membrane thickness *L*.

In the Nielsen model, it is defined that the fillers are impenetrable to a diffusing gas molecule [13]. This assumption leads, in a first approximation, to two considerations.

First, the effective diffusion path through the polymer membrane $L_{eff}$ is expressed according to

(7) $$L_{eff}=\tau \;L$$

where the tortuosity factor τ is a proportionality factor based on the assumptions that the gas molecules cannot penetrate the filler particles, which leads to a longer tortuous path for the permeating molecules. The effective diffusion path is estimated as follows [13,27]: each filler particle contributes to the elongation of the diffusion path by w/2 on average. The expected value $\langle N\rangle$ for the number of filler particles that the permeating gas molecules encounters is estimated with

(8) $$\langle N\rangle =\frac{L}{b}\;\phi_{f}$$

where $\phi_{f}$ is the volume fraction of the fillers and *b* is the thickness of the filler particles (see Figure 2). Therefore, the effective diffusion path $L_{eff}$ can also be expressed as

(9) $$L_{eff}=L\;\left(1+\frac{w}{2\;b}\;\phi_{f}\right)$$

and comparison of Equation (9) with Equation (7) gives

(10) $$\tau =1+\frac{\alpha }{2}\;\phi_{f}$$

with α=w/b as the aspect ratio of the rectangular filler particles.

It is convenient not to change “external” measurable parameters, such as membrane thickness *L* or the pressures at the membrane sides $p_{i}$. With comparison of Equations (6) and (9), an effective diffusion coefficient $D_{eff}$ is calculated with

(11) $$D_{eff}=\frac{D_{0}}{1+\frac{\alpha }{2}\;\phi_{f}}$$

instead of an effective diffusion path $L_{eff}$, where $D_{0}$ is the diffusion coefficient of a continuous homogeneous polymer matrix without fillers.

Second, since the fillers are assumed to be impenetrable, the gas molecules can only be dissolved in the polymer matrix. Therefore, the effective solubility coefficient $S_{eff}$ is calculated with

(12) $$S_{eff}=S_{0}\;\left(1-\phi_{f}\right)$$

where $S_{0}$ is the solubility coefficient of a continuous homogeneous polymer matrix. The Nielsen model can then be expressed as the relation of an effective flow $F_{eff}$ through a filled polymer to a flow $F_{0}$ through the continuous homogeneous polymer matrix while considering Equations (6), (11) and (12) with

(13) $$\frac{F_{eff}}{F_{0}}=\frac{D_{eff}\;S_{eff}}{D_{0}\;S_{0}}=\frac{Pe_{eff}}{Pe_{0}}=\frac{1-\phi_{f}}{1+\frac{\alpha }{2}\;\phi_{f}}$$

where $P_{eff}$ and $P_{0}$ are the permeation coefficients of the filled and homogeneous polymers, respectively.

## 3. Derivation of the Channel Model

As can be seen in Equation (13), only two parameters, the aspect ratio α and the filler volume fraction $\phi_{f}$, are used in the Nielsen model. For numerical simulations, additional parameters are necessary to sufficiently describe the geometric structure of the permeation problem. For the Nielsen model, the fillers are arranged in a regular pattern: rows of rectangular fillers with the longer edge perpendicular to the direction of diffusion, each row offset from the previous one so that the fillers are in the middle of the gaps of the previous row. Therefore, only two more parameters are needed to fully describe the geometric structure: the slit distance *s*, which defines the width of the gap in a row, and the filler distance *d*, which defines the distance between the symmetry axes of two adjacent rows (see Figure 2).

**Figure 3:**
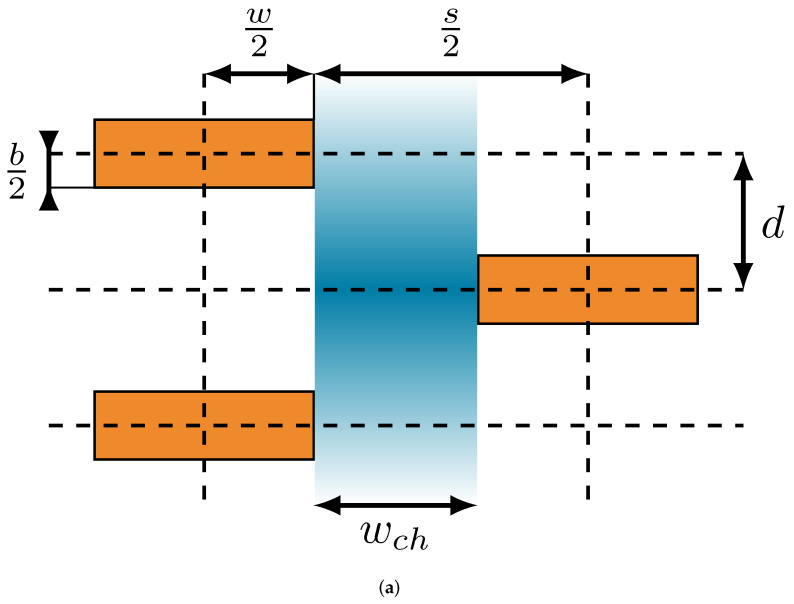

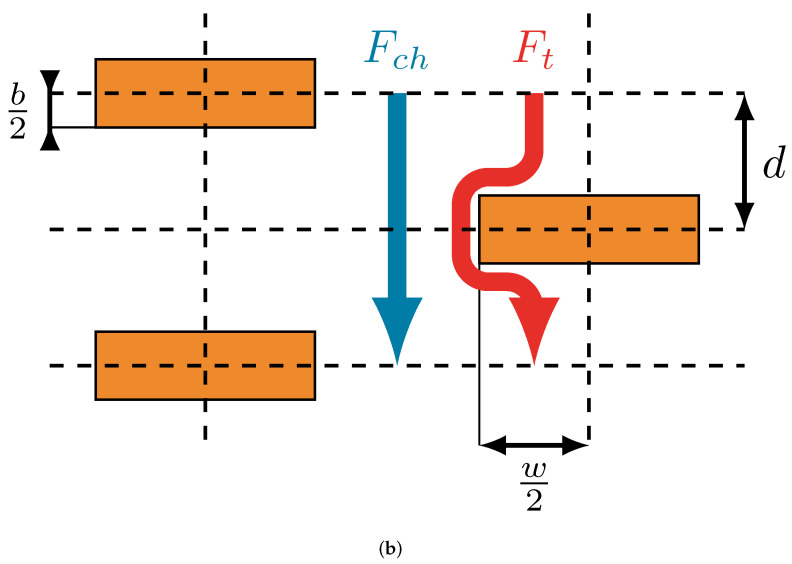
Model sketches. (**a**) Sketch of unit cells (enclosed with dashed lines) with a channel (blue) through the filler rows in which diffusion takes place unhindered. (**b**) Sketch of two superimposed flows in a unit cell: the unhindered flow through the channel $F_{ch}$, and the tortuous flow around fillers $F_{t}$.

Nielsen predicted cases where channels formed in the filler arrangement that would pass through the whole membrane [13]. Therefore, we assumed that permeation through filled polymers is a superposition of two flows: one flow $F_{ch}$, which is unhindered by fillers because it takes place in “channels” that naturally form in regular patterns where the slit distance *s* is larger than the filler width *w*, and another flow $F_{t}$, which follows a tortuous path around the fillers (see Figure 3). This assumption, together with geometrical considerations about unit cells in the filled polymer matrix, allows the calculation of the two missing parameters, filler distance *d* and slit distance *s*.

For the quantitative description of the filler distance *d*, it is necessary to subdivide the filled polymer, similar to the approach of Minelli et al. [29], into defined rectangular unit cells whose two opposite corners are located, respectively, in the centers of the diagonally adjacent filler particles of two neighboring filler rows (see Figure 3). Since the filled polymer matrix is described with a 2D model, all spatial definitions are reduced by one dimension. In a first step, the area of a unit cell is expressed as

(14) $$A_{u}=\frac{w+s}{2}\;d$$

and a unit cell contains the filler area

(15) $$A_{f}=\frac{w\;b}{2}.$$

The filler volume fraction $\phi_{f}$ is expressed as the ratio of the two areas with

(16) $$\phi_{f}=\frac{A_{f}}{A_{u}}=\frac{w\;b}{d\;(w+s)},$$

which is converted to the following form to calculate the filler distance *d*:

(17) $$d=\frac{w\;b}{\phi_{f}\;\left(w+s\right)}.$$

According to Equation (17), the filler distance is also dependent on the slit distance *s*, which is calculated by calculating the ratio between the superposition of the two flows $F_{ch}$ and $F_{t}$ and a flow that represents unhindered permeation in an unfilled polymer matrix, inside two consecutive unit cells (see Figure 3). Since the channels pass through the polymer matrix without interruption, provided that the fillers are arranged regularly and periodically, the flow $F_{ch}$ is unhindered for the whole thickness of the membrane. Therefore, the fraction of unhindered flow in the membrane is only dependent on the width of the channels $w_{ch}$, which is calculated with

(18) $$w_{ch}=\frac{s-w}{2},$$

relative to the width of the unit cells, which leads to the fraction of unhindered permeation

(19) $$f_{ch}=\frac{F_{ch}}{F_{0}}=\frac{s-w}{w+s}.$$

The fraction of the tortuous flow $F_{t}$ can be determined similarly but with two additional conditions: first, only the upper border of the unit cell without a filler particle of width w/2 is considered for the path of $F_{t}$. On the one hand, the filler particle closes off any flow besides the filler channel and, on the other hand, this prevents multiple considerations of $F_{t}$ between other constellations of consecutive unit cells. Second, since the flow is tortuous, its effective path length on the average inside a single unit cell is calculated with

(20) $$L_{u}=d+\frac{\int_{0}^{\frac{w}{2}}z\;dz}{\frac{w}{2}}=d+\frac{w}{4}$$

where the integral in the equation calculates the average of the additional path length *z* caused by the respective filler particle. Using Equation (20) in combination with Equation (7) results in

(21) $$\tau_{t}=1+\frac{w}{4\;d}$$

for the tortuousity of the effective path length. Since two unit cells are required for the flow to return to its initial state (see Figure 3), the fraction for the tortuous path has to be divided by 2 to ensure that each contribution to this flow is counted only once. Taking into account all these assumptions, as well as the width of the unit cells, leads to

(22) $$f_{t}=\frac{F_{t}}{F_{0}}=\frac{1}{2\;\tau_{t}}\;\frac{w}{w+s}$$

for the fraction of tortuous permeation. With Equations (17) and (21), Equation (22) finally becomes

(23) $$f_{t}=\frac{2\;w\;b}{\phi_{f}\;{\left(w+s\right)}^{2}+4\;b\;\left(w+s\right)}.$$

The sum of the fraction of the unhindered permeation $f_{ch}$ and of the tortuous permeation $f_{t}$, respectively, also results in the relative permeation through the filled polymer and can be equalled to Equation (13), resulting in

(24) $$\frac{F_{eff}}{F_{0}}=\frac{1-\phi_{f}}{1+\frac{\alpha }{2}\;\phi_{f}}=f_{ch}+f_{t}.$$

When Equations (19) and (23) are substituted into Equation (24), the result is a quadratic equation for the slit distance *s*. Since *s* is a physical distance, only the positive root of the equation is a reasonable solution. An extensive simplification then leads to

(25) $$\begin{array}{cc} s & =w\;\cdot \;\left[-\frac{2\;\alpha \;\phi_{f}+4}{\phi_{f}\;\alpha \;\left(\alpha +2\right)}\right.\\ \; & +\frac{\sqrt{\alpha^{4}\;\phi_{f}^{2}+6\;\alpha^{3}\;\phi_{f}+4\;\alpha^{2}\;\phi +12\;\alpha^{2}+24\;\alpha +16}}{\phi_{f}\;\alpha \;\left(\alpha +2\right)}]. \end{array}$$

With Equations (17) and (25), the missing parameters from the Nielsen model, filler distance *d*, and slit distance *s* are fully determined, provided that at least one of following conditions is satisfied, which ensure that no collisions between filler particles occur:

(26) $$\begin{array}{cc} s & >w\\ \; & \mathrm{or}\\ d & >b. \end{array}$$

## 4. Numerical Test Setup for the Channel Model

**Figure 4:**
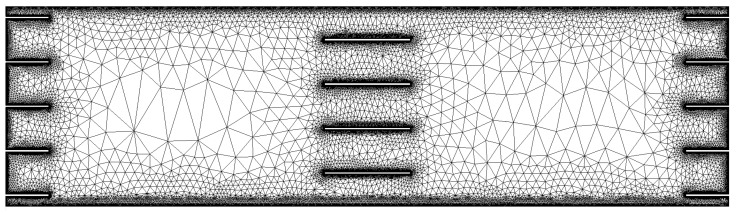
Example of a mesh for the numerical test setups. Dirichlet boundary conditions are defined at top and bottom edges of the mesh, while a periodic boundary condition is defined for left and right edges. The fillers are indicated by rectangular ’holes’ in the mesh, with only one filler per filler row. To improve visibility, the example contains only seven filler rows compared to the 100 filler rows in the numerical test setups.

To verify that the channel model gives reasonable results according to the assumptions of the Nielsen model, 2D geometries of filled polymer matrices were generated in which the fillers were regularly and periodically arranged. The 2D geometries were created and meshed with the free software SALOME [34], using Python macros to generate the filler arrangements. The area size of the geometry depended on the filler width *w*, the slit distance *s*, and the filler distance *d*, since each filler array contained exactly 100 filler rows with one filler each. The fillers were arranged periodically in each row, so that the slit distance spacings between fillers and the fillers cut off at one boundary parallel to the diffusion direction were continued at the opposite boundary. The definition of periodic boundaries also allowed a very small number of fillers per row and thus significant savings in computational power without compromising the accuracy of the simulations (see Figure 4, for example).

The thickness of the fillers *b* was chosen to be similar to the graphene flakes studied by Scherillo et al. [35] with 3 nm, while the aspect ratios that defined the corresponding filler width *w* of the fillers were chosen according to the recent literature (e.g., [23,35,36,37,38,39,40]) with 50, 150, 300, 600, and 900. The goal was to perform simulations that were as close as possible to real experiments. Slit distance *s* and filler distance *d* for each of the regular filler arrangements were calculated and defined according to Equations (17) and (25).

The geometries were more finely meshed around fillers and boundaries, with the impenetrable fillers represented as unmeshed holes. The meshes generated with SALOME were 3D and were converted to 2D with the Python package “meshio” [41]. The meshes thus prepared were then imported into the free FEM solver FreeFEM++ [42]. Periodic boundary conditions were defined for the edges of the geometry, which were parallel to the diffusion direction, while Dirichlet boundary conditions were defined for the other two edges with 1 bar and 0 bar on the upstream and downstream side of the 2D geometry, respectively.

In FreeFem++, Equation (5) was solved to obtain the concentration distribution within the membrane for steady state. The gradient of the solution was averaged over the downstream edge of the geometry with

(27) $$\bar{\nabla C}=\frac{1}{l_{d}}\;\int_{l_{d}}\nabla C\;n_{l_{d}}\;dx$$

where $l_{d}$ is the length of the edge on the downstream side and $n_{l_{d}}$ is the unit vector normal to the edge. Since the diffusion coefficient $D_{0}$ and the solubility coefficient $S_{0}$ were assumed to be constant for the polymer matrix, they cancel out when the permeation coefficient of the filled polymer $Pe_{eff}$ is considered only relative to the permeation coefficient of the unfilled polymer $P_{0}$. For this reason, arbitrary constants were chosen for $D_{0}$ and $S_{0}$. Based on Equations (1), (3), and (27), the relative permeation for each 2D geometry was then calculated with

(28) $$\frac{Pe_{eff}}{Pe_{0}}=\frac{{\bar{\nabla C}}_{filled}}{{\bar{\nabla C}}_{\mathrm{unfilled}}}.$$

## 5. Results and Discussion

**Figure 5:**
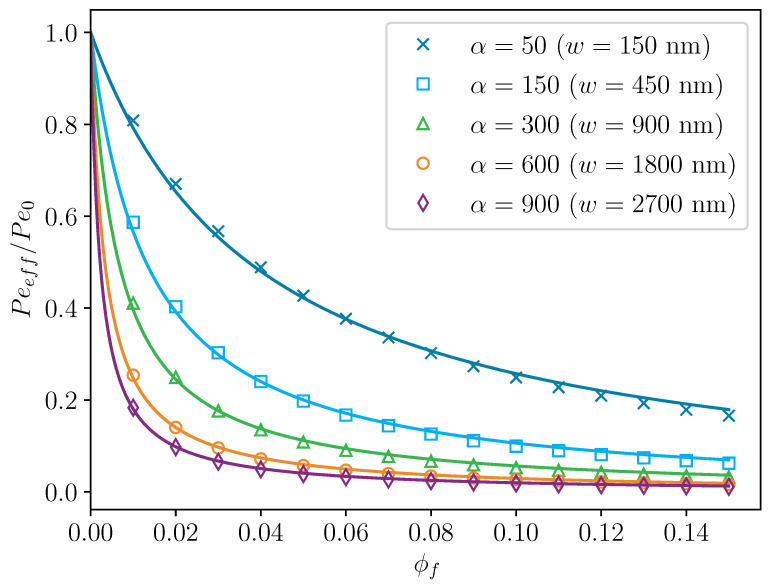
Comparison of predictions of the Nielsen model and the 2D filler simulations augmented with the channel model. The markers represent the simulations while the solid lines in the same color represent the model predictions. The thickness of the fillers *b* was always 3 nm.

Figure 5 shows a comparison between the predictions of the Nielsen model and the 2D filler simulations performed as described in Section 4, calculated with aspect ratios of significantly different magnitudes over the range of filler volume fractions from 0 to 0.15. The residuals between model prediction and simulation results display a correlated behavior, indicating that there are still some effects that are not included in the channel model [43]. Nevertheless, the agreement between model prediction and simulations appears to be sufficient enough to validate the findings with the channel model for the 2D geometries.

**Figure 6:**
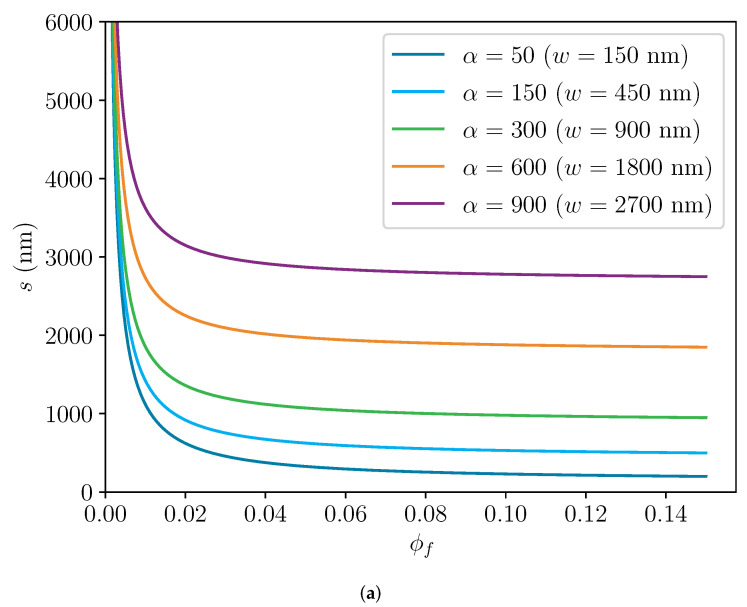

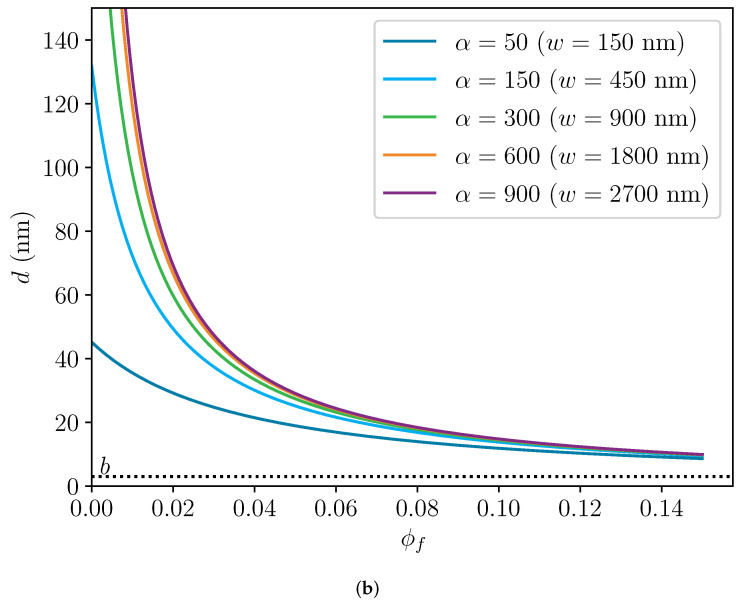
Calculations of slit distance *s* and filler distance. The thickness of the fillers *b* was always 3 nm (dotted line). (**a**) Slit distance *s*, which was calculated with the channel model over the filler volume fraction $\phi_{f}$. (**b**) Filler distance *d*, which was calculated with the channel model over the filler volume fraction $\phi_{f}$.

Figure 6 displays the results the channel model for slit distance *s* and filler distance *d* as a function of the filler volume fraction $\phi_{f}$ according to the predictions of the Nielsen model in Figure 5. After a sharp decline below $\phi_{f}=0.02$, the decrease in the slit distance *s* reduces strongly until it falls below the respective filler width *w* at ϕ=0.75, independent of the aspect ratio α. In contrast, the filler distance *d* approaches a finite value at $\phi_{f}=0$. After substituting Equation (25) into Equation (17), this finite value, which seems to be caused by the fast approach of Equation (25) to infinity, can be approximated with good accuracy by

(29) $$d\left(\phi_{f}=0\right)\approx \frac{b\;\alpha }{2\;\sqrt{3}}$$

where it was assumed that α≫1. Due to the non-realistic result for *d* at $\phi_{f}=0$, the channel model should not be used at filler volume fraction ranges with large slopes of *s*. It is difficult to determine the value of filler volume fraction below which the channel model loses its validity, since no other requirement of the model is violated. Nevertheless, it is assumed that the channel model will produce valid results in a range of $\phi_{f}$ larger than approximately 0.01. In this filler fraction range, the slit distance *s* changes only moderately, while permeation behavior seems to be strongly dependent on filler distance *d*, which should correlate to a mean free path length in which permeation is not disturbed by fillers.

Similar to the slit distance *s*, the filler distance *d* decreases monotonically with decreasing slope and falls below the filler thickness *b*. From Equations (17) and (25), the filler volume fraction $\phi_{f}$ where d=b can be calculated as follows:

(30) $$\phi_{f}\left(d=b\right)=\frac{\alpha^{2}+\alpha -2}{2\;\alpha \;(\alpha +3)}\approx 0.5,\;\mathrm{for}\;\alpha \gg 1.$$

Therefore, the channel model is at least valid for the range of the filler volume fraction of $0.01≲\phi_{f}≲0.5$. Both conditions in Equation (26) are satisfied and *s* does not change rapidly. In this range, the augmentation of the Nielsen model with the channel model provides information about the filler geometry in a polymer membrane, which can then be verified with experimental methods.

## 6. Conclusions and Outlook

Although the Nielsen model is one of the earliest models for permeation in filled polymers, it is still one of the most widely used in the literature. However, the model does not provide quantitative information on arrangements of fillers inside a polymer matrix. For this reason, the channel model was developed in this work, which extends the Nielsen model by determining the relative distances, slit distance *s*, and filler distance *d*, between the fillers in polymer matrices. To our knowledge, this allows, for the first time, for the Nielsen model to relate the permeation properties of filled polymer membranes to the geometric properties of the filler arrangement in simulations and experimental measurements. Simulations with geometries defined according to the Nielsen and the channel models showed good agreement with the predictions of the Nielsen model. This demonstrated that the channel model can be a valuable tool for predicting at least mean geometric distances in studied polymer membranes. Such values can either be good starting values for permeation simulations or used for comparison with experimental results.

The validity range of the channel model was limited to an interval of the filler volume fraction $0.01\leq \phi_{f}\leq 0.5$. This validity range was set solely on the basis of theoretical considerations, since experimental verification is expected to take a long time, if at all possible. This is due to the complexity involved in producing suitable samples and performing measurements, such as permeation experiments and X-ray diffraction, which are necessary to evaluate the geometrical predictions of the channel model. In addition, the channel model as the auxiliary model is limited to the validity range and assumptions of the Nielsen model, which only gives accurate results for lower filler volume fractions $\phi_{f}$ and regular filler arrangements. Moreover, the relationship between a specific effective flow $F_{eff}$ through a membrane and filler arrangements with, respectively, different slit distance *s* and filler distance *d* can possibly be ambiguous. This uncertainty can only be resolved by the elaborate experimental work mentioned before, since only one of these filler arrangements can be found with the channel model.

The channel model was developed explicitly for the Nielsen model, where impermeable fillers oriented perpendicularly to the diffusion direction are assumed. Similar to this work, it will be tested how well the channel model will predict the slit distance *s* and filler distance *d* for the Nielsen model modified by Bharadwaj [15], where different orientations for fillers in regular arrangements are also considered. It is expected that the results from such comparisons will allow further evaluation of the channel model.

## Figures and Tables

**Table 1:** Exemplary overview of common analytical permeation models. This overview of the models is a summary based on the information in [11,12,25,26,27].

Model	Properties	Disadvantages
Maxwell [17]	tortuosity model based on filler volume fraction $\phi_{f}$	2D, only spherical fillers (no aspect ratio), no consideration of distances in filler arrangements, no filler overlapping
Bruggeman [18]	valid for higher filler volume fraction than Maxwell model	2D, only spherical fillers (no aspect ratio), no consideration of distances in filler arrangements, no filler overlapping
Nielsen [13]	tortuosity model based on filler volume fraction $\phi_{f}$ and aspect ratio α	2D, no consideration of distances in filler arrangements, only regular filler arrangements, orientations of fillers only perpendicular to diffusion direction, no filler overlapping
Aris [19,25]	3D, tortuosity model based on filler volume fraction $\phi_{f}$, aspect ratio α, slit distance *s* and filler distance *d*	assumes small *s*, which can lead to overestimation of barrier properties at low $\phi_{f}$
Cussler [14]	tortuosity model based on filler volume fraction $\phi_{f}$ and aspect ratio α, considers regular and random arrangements for fillers	2D, no consideration of distances in filler arrangements, no filler overlapping
Bharadwaj [15]	extents Nielsen model by specific and random filler orientations relative to diffusion direction	2D, no consideration of distances in filler arrangements, only regular filler arrangements, no filler overlapping

## Data Availability

Please find the numerical results and the FreeFem++ script file in the Mendeley repository (published on 29 June 2022): https://doi.org/10.17632/sds582pf6k.1, (accessed on 11 August 2022). If additional data are required, please contact the corresponding author.

## Abbreviations

The following abbreviations are used in this manuscript:

*C*	Permeate concentration
*D*	Diffusion coefficient
*S*	Solubility coefficient
*F*	Permeation flow
Pe	Permeation coefficient
*t*	Independent variable for time
*x*	Independent variable for space (1D)
*V*	Volume
$p_{i}$	Boundary condition for pressures at either side of the membrane
*L*	Thickness of membrane
τ	Tortuosity
$L_{eff}$	Effective thickness of membrane
$D_{eff}$	Effective diffusion coefficient
$S_{eff}$	Effective solubility coefficient
$Pe_{eff}$	Effective permeation coefficient
$S_{0}$	Solubility coefficient of unfilled polymer
$D_{0}$	Diffusion coefficient of unfilled polymer
$Pe_{0}$	Permeation coefficient of unfilled polymer
$F_{0}$	Permeation flow through unfilled polymer
$F_{eff}$	Effective permeation flow through filled polymer
$L_{u}$	Effective thickness in a unit cell inside a filled polymer
$\phi_{f}$	Filler volume fraction in a polymer
*w*	Width of a single filler
*b*	Thickness of a single filler
*d*	Distance between adjacent filler rows in regular filler arrangement
$f_{i}$	Fraction of effective flows over flow in unfilled polymer
*s*	Slit distance between fillers in a single filler row of a regular filler arrangement
α	Aspect ratio of fillers w/b
$l_{d}$	Downstream edge of a rectangular 2D diffusion geometry
$n_{l_{d}}$	Normal vector on $l_{d}$
$\langle N\rangle$	Expected number of interactions of permeate with fillers in polymer membrane
